# Does Cerebral Hemispheric Laterality Control Swallow Performance?

**DOI:** 10.1155/2017/8762610

**Published:** 2017-11-29

**Authors:** Radish Kumar Balasubramanium, Thejaswi Dodderi, Jayashree S. Bhat

**Affiliations:** ^1^Department of Audiology and Speech Language Pathology, Kasturba Medical College, Manipal Academy of Higher Education, Mangalore, Karnataka, India; ^2^Department of Audiology and Speech Language Pathology, Nitte Institute of Speech and Hearing, Mangalore, Karnataka, India

## Abstract

**Objectives:**

It is well established that the brainstem regulates the act of swallow. However, the role of cortex and its influence on swallowing are still a question. Hence, the present study aimed to investigate if cerebral hemispheric laterality controls swallow activity.

**Methods:**

Thirty normal right handed participants were subjected to time test of swallow using 100 ml of water. Dual paradigm was used to investigate hemispheric laterality for swallowing which involved listening to the speech or music stimuli presented binaurally while swallowing. The clinician measured total time taken and hyolaryngeal movement simultaneously which was used to calculate volume/time, volume/swallow, and time/swallow on an offline basis.

**Results:**

Results revealed that swallow performance decreased with the dual task paradigm compared to baseline swallow. These results are suggestive of cortex playing a role during swallowing in the dual task paradigm. Moreover, quantitative parameters like volume/swallow and volume/time were affected more when speech was competing with swallowing. However, music exerted greater interference over the speech for time/swallow.

**Conclusions:**

These results suggests that there exists differential cue lateralization hypothesis which means volume related parameters are controlled by left hemisphere and time related swallowing parameters are controlled at the right hemisphere.

## 1. Introduction

Swallowing is a sensory-motor act that is regulated majorly by the brainstem [1]. However, various studies also have reported that cerebral cortex is involved in triggering the swallow mechanism [2]. This suggests that this task needs high degree of sensorimotor integrity in the cerebral cortex. This viewpoint has led many researchers to contemplate on whether there is a hemispheric laterality for swallowing skills.

Techniques to evaluate the asymmetry for swallowing can be classified into three broad categories. They include brain imaging techniques such as computerised topography [2], Positron emission test [3], and functional magnetic resonance imaging [4, 5]. Secondly, the brain stimulation techniques like transcranial stimulation can be used to map the cortical sites for swallowing. The last one is the dual task paradigm, which uses behavioural responses to measure the cerebral asymmetries. Though each technique has its boon, disadvantages like lack of temporal sensitivity [6], lack of resolution, radiation exposure [7], and risk of seizures in transcranial stimulation [8] lead to many clinical modifications. These banes were however nil in the noninvasive, unimanual task of dual task paradigm [9] and hence it is widely used in many cognitive and psychological experiments. This is an interference task based on the analogy that if language or motor systems share the same neural substrates for swallowing then there will be a decrease in the dual task performance. This is performed by comparing the quantitative data of the isolated task performance with the dual task paradigm.

Various theoretical reasons have been put forward to understand how interference tasks like the dual task paradigm affect swallow performance. They include capacity sharing, cross-talk, and bottleneck. The capacity sharing postulates that reduced performance in the dual task is due to shared neural substrates for a function [10] leading to competition between the tasks whereas the cross-talk deals with decreased performance in tasks due to sharing of similar processing units like attention [11] and memory [12]. Lastly the bottleneck effect reviews reduced performance due to bottleneck resulting from the competition for the same information processing [13].

Many neuroimaging studies have been conducted till date that support the lateralization for swallow. In their positron emission tomography study on eight right handed subjects, Hamdy et al. reviewed that there exists cerebral dominance for swallow only for few sites. The sites include bilateral representation in the caudolateral sensorimotor cortex region. However, unilateral representation was also evidenced in left hemisphere in amygdala, mesial premotor cortex, and dorsal brainstem, with the increased regional cerebral blood flow in the region of right hemisphere anterior insula and orbitofrontal and temporopolar cortices [3]. These were further supported by Mosier et al. who performed functional magnetic resonance imaging on eight healthy adults. On dry and liquid swallows they evidenced that left hemisphere dominance was more than the right, but the patterns were inconsistent [5].

Daniels et al. investigated cortical representation of swallow using the modified dual task paradigm. The tasks considered were finger tapping and silent word repetition during swallowing. They measured laterality of swallowing by comparing the baseline swallow performance with the dual task paradigm swallow performance, on which there was significant decrease in finger tapping rate and volume/swallow during the dual task paradigm. The study concluded that left hemisphere plays an important role during swallowing [14]. However, the study received critical reviews which questioned the reliability of the subjects performing the silent word repetition [15] and no use of right hemisphere dependent tasks [14]. It was also put forth by Kelly and Huckabee that there existed large discrepancy in statistical analysis of standard deviation due to pooling of all data collected from the participants [15]. In contrast to unilateral representation, study by Hamdy et al. reported that there is bilateral representation of swallow with interhemispheric difference for pharynx and oesophagus [16]. However studies have also reported no association between the site of lesion and symptoms of dysphagia [17]. Hence it can be summarized that hemispheric lateralization of swallowing is controversial in review and contradictory in findings and hence the present study was undertaken with the different dual task paradigm where swallowing happens along with either speech or music.

It is well established that speech and music are processed differently in the brain; that is, speech and music stimuli are processed in left and right hemisphere, respectively [18–20], and hence these stimuli were chosen in the present study for the dual task paradigm. It is hypothesized that there would be decrement in the swallowing performance when it happens along with speech or music. Also it is hypothesized that if the swallowing performance decreases significantly more with the speech stimuli in comparison to music stimuli, we can presume that swallowing is controlled by the left hemisphere as speech is controlled by left hemisphere. Thus the present study uses this interference based dual task paradigm to investigate if right and/or left hemisphere controls the swallowing performance.

## 2. Method

### 2.1. Participants

Total of 30 participants in the age range of 18 to 25 years (mean age of 21.5 years) participated in the study. All the participants were right handed as determined by the Edinburgh Handedness Inventory [21] and cognitive dysfunction was ruled out using Mini Mental State Examination [22]. All the participants were free from speech, language, and neurological problems and had no history of any surgery done to the oropharyngeal apparatus.

### 2.2. Test Material

The test stimuli consisted of speech and music. The speech stimuli were lyrics of the English song “Waving Flag” recorded using Praat software (Version 5.2.23) [23] by a female speaker in a neutral intonation pattern, which was sampled at 44 kHz and saved in  .wav format. Music stimulus used was a classical music instrumental of flute, tabla, and saxophone.

### 2.3. Procedure

The participants were seated comfortably in a straight back chair and completed the timed test of swallow. The testing was done across two conditions, the baseline condition and dual task paradigm condition. In the baseline condition, all participants were given 100 ml of water in a cup and were instructed to drink quickly and continuously with no spillage. And, for the dual task paradigm, the music stimuli were presented binaurally using Logitech headphones at 60 dBSPL. Instructions given were to concentrate on the auditory stimuli while performing the timed test of swallow simultaneously. The same procedure was then followed using the speech stimuli. After the swallow task, the clinician performed a visual inspection of the cup to ensure that there was no water residue.

For every swallow performance, the clinician simultaneously measured two online parameters. Firstly the total time taken by the participant to swallow was measured using a digital stopwatch. The timer was started when the participant placed the cup on the lower lip to drink and ceased with removal of the cup from the lips. Secondly, the total number of swallows was calculated by visualizing the number of hyolaryngeal movements during the swallow task. After the dual task paradigm, two questions related to the music and speech stimulus were asked to the participant to confirm alertness to the auditory stimuli during the dual task. Trials with 50% wrong answers or significant water residue in the cup were not considered and were subjected to retesting. The offline data was used to calculate volume/swallow, volume/time, and time/swallow. A total of 90 tokens (30 subjects × 3 swallows) were collected from all participants with each session lasting 10 minutes.

### 2.4. Statistical Analysis

The data was subjected to statistical analysis using SPSS (Version 17). The mean and standard deviation values were calculated and tabulated for all participants using descriptive statistics. One-way ANOVA was performed to note statistical significance between three performances (baseline, speech, and music) across swallow tasks. Multiple comparisons using Bonferroni's post hoc test were also performed to see significant differences across the conditions. *p* value less than 0.05 was considered for statistical significance.

## 3. Results

The present study investigated the hemispheric laterality for the swallowing performance using modified dual task paradigm. Descriptive statistics was conducted to obtain mean and standard deviation (SD) which are in Table 1.

From Table 1, it is evident that there was a decrease in the mean values for volume/swallow and volume/time for dual task paradigm condition when compared to the baseline condition. These results suggest that in the presence of a competing signal, that is, speech and music, the swallow performance decreased. Moreover, quantitative parameters like volume/swallow and volume/time were affected more when speech was competing. This is suggestive of higher degree of interference being exerted by speech signal over music condition for volume related swallow parameters. However, there was an increase in the mean values of time/swallow when compared to baseline condition. It is also observed that this increase was more when music was competing in comparison to the case when speech was competing. This suggests that right hemisphere exerts greater interference over the speech for time/swallow.

The results of one-way ANOVA collectively suggest significant main effect for the tasks (*F* = 8.310 at *p* ≤ 0.001 in baseline condition, *F* = 1.441 at *p* = 0.242 in the music condition, and *F* = 18.274 at *p* ≤ 0.001 in the speech condition) at *p* < 0.05. Multiple comparisons across the three conditions were performed using Bonferroni's post hoc analysis. Collectively, there was a statistical significant difference across all the conditions for volume/swallow and volume/time at *p* < 0.05. Volume related parameters such as volume per swallow and volume per time decreased when speech stimuli were presented compared to music stimuli presentation. However, time/swallow did not exhibit any significant difference across the three conditions at *p* > 0.05 although time per swallow increased slightly when music stimuli were presented.

## 4. Discussion

The present study was undertaken to investigate if right or left hemisphere controls swallow performance using the modified dual task paradigm. Results revealed that swallow performance decreased with the dual task paradigm when compared to the baseline swallow. These results are suggestive of the cortex playing a role during swallowing [2] in the interference task. Moreover, quantitative parameters like volume/swallow and volume/time decreased when speech stimuli were presented binaurally in the dual task paradigm compared to the case when music stimuli were presented. This provides evidence on the left hemisphere exerting a neural control over swallow performance compared with right hemisphere. This in turn reflects activation of sensorimotor areas, planning, and execution required for swallow being controlled in the left hemisphere [14] additional to the brainstem role.

The results of our study can also be attributed to differences in processing loads of the stimuli. That is, unlike music the speech stimuli must have maintained a higher degree of memory load [12] and attention load [11]. This acted as interference during swallowing. Hence in presence of the speech stimuli higher processing loading and demand are met by the left hemisphere. So involvement of another task like swallow will be partially inhibited by the left hemisphere leading to decreased performance. These viewpoints clearly highlight the role of hemispheric laterality in swallow function, with left hemisphere dominating compared with the right hemisphere.

The results of the present study are in consonance with the study by Daniels et al. who also support the role of left hemisphere in the initiation of swallow [14]. The results of our study are supportive of the cross-talk theory [10]. This suggests that in presence of speech stimuli higher processing loading and demand have to be met by the left hemisphere. So involvement of another task like swallow was partially inhibited by left hemisphere, leading to decreased performance. That is, as speech and swallowing are controlled by the left hemisphere, there was interference in the swallowing function.

Also, our results point out that there was no significant difference across the three conditions for time/swallow parameter. However, mean values have indicated that there was a greater increase in the time/swallow parameter when music stimuli were presented in comparison to the case when speech was presented. This suggests the role of right hemisphere in the regulation of time related parameters for swallowing. However, this is only a speculation which needs more detailed investigation. More specific measures like reaction time (in msec) probably would offer statistical significance. Results collectively suggest that volume related parameters are controlled by left hemisphere and time related parameters are controlled by right hemisphere. This viewpoint is similar to differential cue lateralization hypothesis [24, 25] where frequency related parameters for speech prosody are processed in right hemisphere and temporal parameters for speech prosody are processed in the left hemisphere. Hence, it can be said that differential cue lateralization exists even for swallowing and is evident in the present study. In a nutshell, it can be concluded that left hemisphere is dominant for quantity related parameters like volume and right hemisphere for temporal parameters.

## 5. Conclusion

The present study was designed to investigate if right or left hemisphere controls swallow performance for which the dual task paradigm was administered among 18 to 25 years healthy adults using the timed test of swallow. Results revealed decreased mean values for volume per swallow and swallowing capacity in dual task paradigm condition in comparison to the baseline condition. However, there is no significant difference for time per swallow parameter between the baseline condition and dual task paradigms using speech and music. It can be concluded that left hemisphere is dominant for quantity related parameters like volume and right hemisphere for temporal parameters like time. Further research is warranted to investigate similar phenomena across individuals with dysphagia to further support the findings.

## Figures and Tables

**Table 1:** Mean and standard deviation (SD) values of time test of swallow across three conditions.

Tasks	Volume/swallow (ml)		Volume/time (ml/sec)		Time/swallow (sec)	
	Mean	SD	Mean	SD	Mean	SD
Baseline	17.89	5.51	15.59	4.24	1.09	0.26
Music	14.06	4.91	11.92	3.65	1.38	1.10
Speech	12.79	4.68	9.67	3.54	1.30	0.26
